# An Uncommon Finding in an Adult: A Case Report of Hypoplastic Left Heart Syndrome and Protein-Losing Enteropathy

**DOI:** 10.7759/cureus.62705

**Published:** 2024-06-19

**Authors:** Jayasree Ravilla, Siva Naga Yarrrarapu, Sai Rakshith Gaddameedi, Andrew Kruger, Doantrang Du

**Affiliations:** 1 Internal Medicine, Monmouth Medical Center, Long Branch, USA; 2 Gastroenterology and Hepatology, Monmouth Medical Center, Long Branch, USA

**Keywords:** congenital, protein, enteropathy, hypoplastic, fontan

## Abstract

Protein-losing enteropathy (PLE) is a rare disorder with diverse causes, but the treatments are limited and understudied. It is often associated with significant mortality and morbidity. The survival of hypoplastic left heart syndrome (HLHS) in infants without any intervention is usually 4.5 days, and 30-day mortality is 95%. However, with surgical intervention, survival at 20 years is 80%. HLHS can lead to protein-leading enteropathy and is corrected by the three-step procedures (Norwood, Glenn, and Fontan) during infancy. We report a case of Fontan procedure postoperative HLHS associated with PLE and describe its clinical course and outcome. The main intention of reporting this case is to provide awareness among physicians while dealing with refractory cases of hypoproteinemia and appropriate management based on the literature.

## Introduction

Hypoplastic left heart syndrome (HLHS) is a rare and serious congenital heart defect that affects approximately 1 in 3,500 to 1 in 12,500 live births [1]. This condition involves impaired blood flow to the heart due to an underdeveloped left heart that precludes reconstructive surgery (Norwood, Glenn, and Fontan surgery). Protein-losing enteropathy (PLE) involves the abnormal loss of proteins through the GI tract, a phenomenon attributed to various underlying causes. Individuals exhibiting low serum protein levels, after excluding alternative causes of hypoproteinemia, should be considered potential PLE suspects, according to the available medical literature [1-3]. In this case report, we discuss the connection between HLHS and PLE.

## Case presentation

A 39-year-old Caucasian male with a history of congenital HLHS with situs inversus post-Fontan procedure (at age 2), Crohn’s disease involving the terminal ileum (in remission for the past six years), and IV drug use presented to the ER with an opioid overdose treated with 0.4 mg IV Narcan. His vitals showed a heart rate of 66/min, a blood pressure of 100/60 mmHg, and a respiratory rate of 13/min. The patient’s oxygen saturation was in the 70s, so he was intubated for hypoxic respiratory failure, after which saturation improved to the 90s. The physical exam revealed diffuse rhonchi in all lung fields. He was admitted to the ICU for ventilator support.

**Figure 1:**
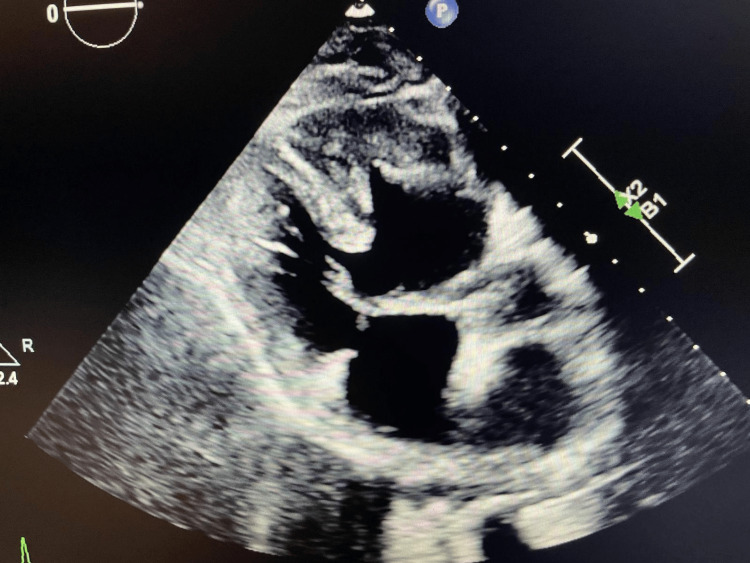
TTE showing hypoplastic left heart post-Fontan procedure and dextrocardia TTE, transthoracic echocardiogram

During admission, his basic laboratory tests remained within normal limits except for his serum albumin level (Table 1). He stayed on ventilator support for four weeks during his hospital stay, experiencing difficulty weaning his FiO2 and failing pressure support trials. The patient’s input and output showed a positive balance of 1 liter despite having adequate renal function and minimal fluid input through orogastric tube feeds. Serum albumin and immunoglobulin levels remained low throughout the admission (Table 2). On hospital day 12, the patient developed generalized edema, crackles, and pitting edema around the extremities. Diuresis with 40 mg IV Lasix daily for five days improved his SpO2 to the mid-90s and decreased his FiO2 to 60%. Albumin infusion was initiated at 2 g/kg/day for five days. Despite these attempts, serum protein levels did not improve. Other causes of hypoalbuminemia, such as chronic liver disease, nephrotic syndrome, and autoimmune etiologies, were ruled out by echo, urinalysis, and negative autoimmune markers (Table 3). A transthoracic echocardiogram (TTE) showed findings of dextrocardia, an unbalanced atrioventricular valve, a small left ventricular cavity with normal wall thickness, and a large ventricular septal defect post-Fontan-Glenn procedure connecting the right ventricle to the left ventricle. The ejection fraction was 35%, unchanged from his previous TTE one year prior (Figure 1).

**Table 1:** Basic labs pertinent to the case Lab results show a complete blood count and metabolic panel on the day of admission versus Day 10 and the last day of admission.

Parameter	On the day of admission	On Day 10	On the day of the transfer
White blood count (normal: 4.50-11 × 10^3^/uL)	5.5 × 10^3^/uL	5 × 10^3^/uL	4.5 × 10^3^/uL
Hemoglobin (normal: 12-15.5 g/dL)	13 g/dL	12.5 g/dL	11.5 g/dL
Platelet count (normal: 140-450 × 10^3^/uL)	200 × 10^3^/uL	198 × 10^3^/uL	168 × 10^3^/uL
Serum sodium (normal: 135-145 mmol/L)	140 mmol/L	139 mmol/L	140 mmol/L
Serum potassium (normal: 3.5-5.2 mmol/L)	3.8 mmol/L	3.5 mmol/L	3.2 mmol/L
Bilirubin total (normal: 0.21-2 mg/dL)	1 mg/dL	1.5 mg/dL	1.2 mg/dL
Alanine aminotransferase (normal: 10-43 U/L)	45 U/L	55 U/L	50 U/L
Aspartate aminotransferase (normal: 13-41 U/L)	40 U/L	39 U/L	38 U/L
Alkaline phosphatase (normal: 42-119 U/L)	78 U/L	90 U/L	95 U/L
Serum creatinine (normal: 0.6-1.2 mg/dL)	1.1 mg/dL	0.9 mg/dL	1.1 mg/dL
Blood urea nitrogen (normal: 5-21 mg/dL)	19 mg/dL	20 mg/dL	15 mg/dL

**Table 2:** Serum protein levels Lab results show serum albumin and Ig levels on Day 1 versus Day 10 and post-albumin infusion.

Serum proteins	On the day of admission	On Day 10	Levels after albumin infusion
Albumin (normal: 3.4-5.4 g/dL)	2.5 g/dL	1.8 g/dL	1.7 g/dL
IgG (normal: 6-16 g/dL)	6.2 g/dL	6 g/dL	5.9 g/dL
IgA (normal: 0.8-3 g/dL)	1 g/dL	0.7 g/dL	0.7 g/dL
IgM (normal: 0.4-2.5 g/dL)	2.3 g/dL	2.3 g/dL	2.2 g/dL

**Table 3:** Levels of autoimmune markers Lab results of miscellaneous tests pertinent to the case.

Parameter	Value
Urine protein	200 mg
Urine protein creatinine ratio	0.23
Anti-nuclear antibodies	Negative
Anti-citrullinated protein antibodies	Negative
Anti-Jo-1 antibodies	Negative
Rheumatoid factor	Negative

**Figure 2:**
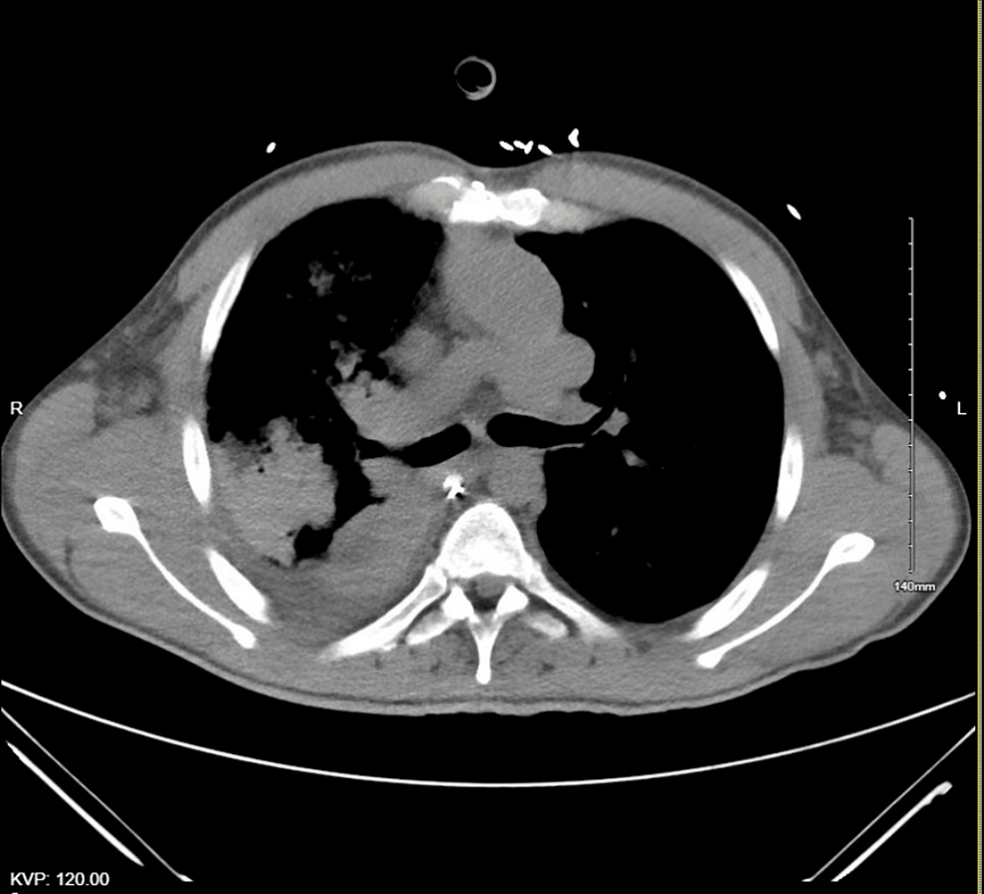
CTPE showing dextrocardia and post-Fontan procedure with the single right ventricle CTPE, computed tomography for pulmonary embolism

A computed tomography for pulmonary embolism was performed due to failure to wean off his ventilator, which showed no embolism. The scan revealed consolidation changes on the right side, along with varus positioning and post-Fontan surgery alterations (Figure 2). He received IV antibiotics with levofloxacin 750 mg for five days to manage right-sided pneumonia associated with the consolidative changes seen on the CT scan. Figure 1 illustrates dextrocardia, postoperative findings, and consolidation in the right lower lobe. Further investigation into the causes of low albumin included calculating alpha-1 antitrypsin (A1AT) clearance at 32 ml/day while on proton pump inhibitor prophylaxis in the ICU. Tests ruled out other GI protein losses, such as GI bleeding and malabsorption disorders, confirmed by negative fecal occult blood and tissue transglutaminase IgA antibodies for celiac disease. Stool infection workup results were unremarkable. Collectively, these diagnostic findings suggest protein loss associated with PLE.

Given the complexity surrounding the treatment of PLE, IV corticosteroids were initiated on Day 17 at 120 mg/day, in addition to as-needed IV diuresis. Clinical signs of fluid overload slightly improved, although there was no change in serum protein levels. Persistent hypoalbuminemia was considered secondary to PLE from the Fontan procedure and heart failure. A decision was made to transfer the patient to a higher-level center specializing in the management of PLE secondary to the Fontan procedure.

## Discussion

PLE is a rare cause of hypoalbuminemia. It is usually caused by the loss of proteins from the GI mucosa through abnormal venous or lymphatic flow [1]. Patients who underwent the Fontan procedure have high central venous pressure, leading to enteric congestion and loss of proteins through the bowel [4]. In humans, PLE is mainly linked to primary intestinal lymphangiectasia, which is caused by genetic predispositions or idiopathic lymphatic blockages [5]. PLE is caused in mainly three main ways: (1) primary ulcerative GI disorders like GI malignancies, carcinoid tumors, IBDs, and erosive ulcers of the stomach and duodenum and infectious causes like *Clostridium difficile* colitis; (2) non-ulcerative GI causes like Menetrier disease, tropical sprue, celiac disease, amyloidosis, eosinophilic gastroenteritis, bacterial overgrowth, intestinal parasitic infections, Whipple disease, collagenous colitis, AIDS, systemic lupus erythematosus, and rheumatoid arthritis; and (3) disorders causing increased interstitial pressure or lymphatic obstruction like primary intestinal lymphangiectasia, right-sided heart failure, constrictive pericarditis, congenital heart disease, Fontan procedure for single ventricle, cirrhosis with portal hypertension gastropathy, hepatic venous outflow obstruction, mesenteric tuberculosis or sarcoidosis, retroperitoneal fibrosis, lymphoenteric fistula, lymphoma, and thoracic duct obstruction [2,6].

Several risk factors for PLE have been identified, including age at Fontan, right ventricular morphology in HLHS, and the presence of pleural effusions. Older age at Fontan operation may contribute to the development of systemic pulmonary collaterals, which can negatively impact the Fontan circulation. Despite advancements in treatment, transplant-free survival rates have not significantly improved in recent years [6,7]. The pathophysiology is complex, and the proposed mechanism includes a combination of altered hemodynamics associated with increased mesenteric vascular resistance and systemic inflammation, along with altered enterocyte basal membrane glycosaminoglycan makeup [7].

The most common symptoms are abdominal pain, edema, bloating, and ascites [8]. The first diagnostic test is a high level of fecal A1AT while excluding other causes of hypoalbuminemia such as malnutrition and liver disease. An A1AT value of greater than 27 ml/day usually reflects GI losses and has a sensitivity of 80%. The other lesser-used alternative diagnostic tests to A1AT are technetium 99 mm labeled human serum albumin scintigraphy and creatinine albumin clearance [8,9].

The treatment is focused on reducing the congestion through medications like diuretics and angiotensin-converting enzyme inhibitors, along with replacing albumin through periodic infusions. Due to GI protein and fat losses, it is recommended to consume a diet rich in proteins (>2 g/kg/day) and medium-chain triglycerides (MCT), as MCT is readily absorbed into the bloodstream, bypassing lymphatics [9,10]. Oral steroids like budesonide have a high enteric anti-inflammatory effect and are considered to be an efficacious treatment after the Fontan procedure. It is also noted through literature that unfractionated subcutaneous heparin can be useful as it acts as a mechanical barrier and decreases the permeability of large molecules like albumin, thus reducing GI losses. The other medical options used include sildenafil, octreotide, and spironolactone. Sildenafil, in particular, is found to improve ventricular function by decreasing pulmonary vascular resistance in post-Fontan patients. In medically refractory cases, surgical repair or transcatheter Fontan fenestration may be considered. Patients who undergo fenestration should be on a full dose of anticoagulation due to the high risk of emboli. The last option would be heart transplantation [10-12].

Treatments varied widely for PLE, and the respondents included a high-protein diet (53%), enteral budesonide (36%), sildenafil (32%), prednisone (12%), IV or subcutaneous heparin (5%), and octreotide (4%). The most responsive treatment was a combination of a high-protein diet and budesonide, which showed results in 26% of patients. We treated our patient with a similar regimen, but due to limited response, he had to be transferred to a different facility.

## Conclusions

The comorbidities associated are conduit-related, cardiac, vascular, Fontan-associated liver disease, lymphovascular changes, and renal disease. Large multicentric studies are essential to establish risk factors involved in its development and to define the efficacy of various treatment modalities previously used for its management.
